# Biofabrication of Cell-Laden Gelatin Methacryloyl Hydrogels with Incorporation of Silanized Hydroxyapatite by Visible Light Projection

**DOI:** 10.3390/polym13142354

**Published:** 2021-07-18

**Authors:** Jimmy Jiun-Ming Su, Chih-Hsin Lin, Hsuan Chen, Shyh-Yuan Lee, Yuan-Min Lin

**Affiliations:** 1 Institute of Oral Biology, School of Dentistry, National Yang Ming Chiao Tung University, Taipei 112, Taiwan; jimmy.su.js@gmail.com; 2 Graduate Institute of Nanomedicine and Medical Engineering, Taipei Medical University, Taipei 110, Taiwan; melodylin@tmu.edu.tw; 3 Department of Dentistry, School of Dentistry, National Yang Ming Chiao Tung University, Taipei 112, Taiwan; tess1994131@gmail.com (H.C.); sylee@ym.edu.tw (S.-Y.L.); 4 Department of Stomatology, Taipei Veterans Hospital, Taipei 11221, Taiwan

**Keywords:** gelatin methacrylate, hydroxyapatite, silanization, mesenchymal stem cells, digital light processing projection, 3D printing

## Abstract

Gelatin methacryloyl (GelMA) hydrogel is a photopolymerizable biomaterial widely used for three-dimensional (3D) cell culture due to its high biocompatibility. However, the drawback of GelMA hydrogel is its poor mechanical properties, which may compromise the feasibility of biofabrication techniques. In this study, a cell-laden GelMA composite hydrogel with a combination incorporating silanized hydroxyapatite (Si-HAp) and a simple and harmless visible light crosslinking system for this hydrogel were developed. The incorporation of Si-HAp into the GelMA hydrogel enhanced the mechanical properties of the composite hydrogel. Moreover, the composite hydrogel exhibited low cytotoxicity and promoted the osteogenic gene expression of embedded MG63 cells and Human bone marrow mesenchymal stem cells (hBMSCs). We also established a maskless lithographic method to fabricate a defined 3D structure under visible light by using a digital light processing projector, and the incorporation of Si-HAp increased the resolution of photolithographic hydrogels. The GelMA-Si-HAp composite hydrogel system can serve as an effective biomaterial in bone regeneration.

## 1. Introduction

Bone tissue engineering aims to restore damaged bone by using a combination of cells, biomaterials, and growth factors [1,2]. Hydrogels are commonly used biomaterials in tissue engineering due to their ability to encapsulate a wide range of cell types and their high water retention [3]. Gelatin is a biocompatible and biodegradable collagen-derived hydrogel that provides the three-dimensional (3D) microenvironment to support cell proliferation and differentiation [4,5]. It exhibits greater solubility and less antigenicity than collagen [6]. However, the limitation of the gelatin hydrogel is its instability at body temperature [7,8]. To solve this problem, numerous methods have been adopted to enhance the stability of gelatin through chemical modification [9]. Functionalization of gelatin with methacrylate groups can generate a covalently crosslinked hydrogel, gelatin-methacryloyl (GelMA), which is more stable than unmodified gelatin hydrogels are [10]. Recently, composite hydrogels have been constructed by incorporating various inorganic nanoparticles including graphene oxide, gold nanoparticles, and carbon nanotubes to improve the mechanical properties in tissue engineering [11,12]. To increase the potential of bone tissue engineering, an optimal hydrogel system should be able to regulate the material properties and encapsulate pro-osteogenic substances [13]. To mimic the in vivo microenvironment, selecting natural inorganic substances from human bone as a reinforcing filler may be a suitable strategy to strengthen GelMA hydrogels.

The major inorganic component of human bones is hydroxyapatite (HAp), which is a type of calcium phosphate salt that is deposited within the protein matrix [14]. HAp can promote new bone formation through osteoconduction without causing toxicity [15,16]. In clinical practice, HAp has been used in orthopedic or dental implants over the past few decades [17,18,19]. Moreover, nanosized HAp powders have drawn attention for their sizable surface area and uniform particle size, which have led to improvements in the mechanical properties and favorable performance in bioactivity [14,20]. However, the clinical use of nanoscale HAp is limited because it is difficult to use when mixed with blood or body fluid, it easily flows away from the origin implant site. Therefore, conjugating HAp with suitable materials to construct a composite hydrogel may improve the localization of HAp and enhance its practical applicability [21,22]. In dentistry, silanization has been widely applied to enhance the bonding between inorganic fillers and organic resins [23,24]. We first hypothesized that the silanization of HAp can improve the mechanical strength of GelMA hydrogels with covalent crosslinking at the interface.

Mesenchymal stem cells (MSCs) are multipotent adult stem cells that can differentiate into bone tissue [25]. Recently, MSCs have been used as a therapeutic material to promote bone regeneration [26], and hydrogels have become a suitable material to carry and localize cells at the fracture site [27]. The mechanical loading of the surrounding microenvironment verifiably directs cell differentiation [28]. A physical cue, especially the matrix stiffness, can be modulated to control the stem cell fate by manipulating the encapsulating hydrogels [29,30]. To construct the physiologically relevant GelMA-based composite hydrogels to encapsulate, MSCs may have the capacity to promote new bone formation, in addition, embedded stem cells can be deposited locally after hydrogel degradation.

Hydrogels are conventionally prepared using molds to create simple structures including a bulk hydrogel or a thin layer. Recently, photolithography has been a prominent microfabrication technique to create geometric patterns with ultraviolet (UV) light on the photo-sensitive materials, and cell-laden GelMA hydrogels have been precisely produced by using photomasks under UV light [31]. The most frequently used photoinitiator of GelMA hydrogels is Irgacure 2959 (CAS: 106797-53-9), which can only be activated by UV light. However, UV radiation may cause cytotoxicity and DNA damage in embedded cells during biofabrication. To avoid the adverse effects of the UV system, the VA-086 photoinitiator (CAS: 61551-69-7) was reported to polymerize GelMA hydrogels by using a blue light-emitting diode (440 nm) in our previous study [32]. The visible light projection method can be used to fabricate hydrogels in a defined pattern and accelerate the process to maintain high cell viability. Digital light processing (DLP) projectors have a superior contrast ratio and excellent grayscale, and they may be a suitable choice to photopolymerize the hydrogel at 1080p resolution. In this study, we aimed to develop a reinforced composite hydrogel and a simple assembly projection method to establish a digital microfabrication procedure for GelMA hydrogels.

Herein, we first used the silanized HAp (Si-HAp) as additives in the hydrogel system and developed a photocrosslinkable GelMA-based composite hydrogel (GelMA/Si-HAp) for photolithography. The surface composition of nanoscale HAp powders was examined by particle size analysis, FT-IR, XPS, and SEM. In addition, the mechanical properties of the composite hydrogels were measured by compression test to evaluate the reinforcing effect of Si-HAp. The biocompatibility and osteogenic differentiation of encapsulated osteoblasts and mesenchymal stem cells were also evaluated. Furthermore, we developed a visible light curing system by a commercial DLP-based projector to micro-fabricate these composite hydrogels into complex architecture.

## 2. Materials and Methods

### 2.1. Synthesis of Gelatin Methacryloyl Hydrogels

Gelatin methacrylate (GelMA) was synthesized from gelatin (Catalog No.: G2500, Sigma-Aldrich, Saint Louis, MO, USA) according to the method reported by Nichol et al. [33]. In brief, 5 g of gelatin was dissolved in 50 mL of phosphate buffered saline (PBS, Catalog No.: 10010023, Thermo Fisher Scientific, Waltham, MA, USA) and heated at 60 °C until gelatin was entirely dissolved. 5 mL methacrylate anhydride (Catalog No.: 276685, Sigma-Aldrich, Saint Louis, MO, USA) was added (at a slow rate of 0.1 mL/min) to the gelatin solution as it was being stirred, and the mixture was allowed to stand and react for 3 h at 50 °C. After 3 h, the reaction was terminated by adding 250 mL of warm PBS (50 °C) and the solution was dialyzed through modified polyethersulfone (PES) hollow fibers (10 kDa molecular weight cut-off, Repligen, Boston, MA, USA) against distilled water at 50 °C for 24 h to remove the methacrylic acid. The solution was lyophilized for 3 days to obtain white sponge-like products and then was stored at −80 °C until further use. The methacrylation level was analyzed using ^1^H-NMR (Ascend 400, Bruker, Billerica, MA, USA).

### 2.2. Silanization of HAp

The Si-HAp was prepared according to a published protocol [34]. Briefly, 5 g of HAp (Catalog No.: 677418, Sigma-Aldrich, Saint Louis, MO, USA), 0.1 g of n-propylamine (Catalog No.: L15808, Alfa Aesar, Tewksbury, MA, USA), and 0.5 g of 3-methacryloxypropyltrimethoxysilane (Catalog No.: A17714, Alfa Aesar, Tewksbury, MA, USA) was added to 100 mL cyclohexane (Catalog No.: 179191, Sigma-Aldrich, Saint Louis, MO, USA). The solution was stirred at 25 °C for 30 min and then heated at 60 °C for an additional 30 min. The solution was transferred into a rotary evaporator at 60 °C to remove the solvent and then heated at 95 °C for 1 h. Finally, the powder was dried at 60 °C in a vacuum oven for 72 h. The elemental composition of silanized hydroxyapatite was obtained using X-ray photoelectron spectroscopy (ESCALAB 250, Thermo Scientific, Waltham, MA, USA). The surface morphology of the Si-HAp was observed through scanning electron microscopy (Catalog No.: JSM-7600F, JEOL, Peabody, MA, USA).

### 2.3. Preparation of GelMA-HAp Composite Hydrogels

GelMA composite hydrogels were obtained by mixing 15% (*w*/*v*) GelMA with 1–3% (*w*/*v*) HAp or Si-HAp powders in the presence of 1% (*w*/*v*) 2,2′-Azobis[2-Methyl-*N*-(2-hydroxyethyl) propionamide] VA-086 photoinitiator (Catalog No.: 013-19342, FUJIFILM Wako Pure Chemical, Richmond, VA, USA) followed by exposure to blue light (440 nm, 8 mW/cm^2^) for 1 min. Before photopolymerization, ultrasonic sonication (20 kHz, Misonix, New York, USA) was performed until the large aggregates fully disappeared. For cell encapsulation, 1 × 10^6^/mL MG63 cells or 1 × 10^7^/mL MSCs were embedded into composite hydrogels. After complete polymerization, the hydrogels were washed twice with PBS and added to the fresh culture medium.

### 2.4. Projection Photolithography of GelMA Composite Hydrogels Using a DLP-Based Projector

A DLP-based projector (Catalog No.: D912HD, Vivitek, Taipei, Taiwan) was fixed vertically on a rack 10 cm above the sample tank. GelMA mixture solution (100 µL) containing 1% VA-086 and 5 × 10^5^ MSCs was evenly spread on the bottom of petri dishes (9.6 mm^2^). The projection images were designed using PowerPoint (Microsoft, Redmond, WA, USA) and the designed patterns were directly projected onto the surface of the cell-suspended GelMA solution. The projected images were black and white. After a projection time of 40 s, PBS was added into the dish and the unpolymerized solution was removed by gentle shaking, and then the hydrogels were incubated in the culture medium. The viability of the encapsulated cells was evaluated after 6 h and after 7 days of incubation.

### 2.5. Characterization of Si-HAp Powders

The functional groups of HAp powders were identified through attenuated total reflection FTIR (ATR-FTIR) analysis (Thermo Fisher Scientific, Waltham, MA, USA). HAp and Si-HAp powders were directly measured with a resolution of 5 cm^−1^ and a total of 16 scans. The spectrum of 600 to 4000 cm^−1^ was examined. The surface elemental composition was analyzed by X-ray photoelectron spectroscopy (ESCALAB 250, Thermo Fisher Scientific, Waltham, MA, USA). The binding energy of the silicone was approximately 104 eV. The nanostructure of dry HAp powders and lyophilized composite hydrogel were examined using SEM.

### 2.6. Mechanical Properties of GelMA Composites

Uniaxial compression measurements were performed using a universal testing machine (Cometech, Taichung, Taiwan) at room temperature at the compression mode. Hydrogel samples were prepared in a polytetrafluoroethylene mold with an internal diameter of 6.4 mm and thickness of 5 mm and photo-crosslinking was performed under blue light exposure (440 nm, 10 mW/cm^2^) for 2 min. All measurements were tested at a crosshead speed of 1 mm/min until the samples were compressed to 50% strain.

### 2.7. Culture of Cells

MG63 cells were maintained in Dulbecco’s modified eagle medium (DMEM, Catalog No.: 12430054, Thermo Fisher Scientific, Waltham, MA, USA) supplemented with 10% fetal bovine serum (FBS, Catalog No.: 10082147, Thermo Fisher Scientific, Waltham, MA, USA) and 1% Penicillin-Streptomycin-Amphotericin B antibiotics (Catalog No.: 15240062, Thermo Fisher Scientific, Waltham, MA, USA). Cells were cultured in a humidified incubator at 37 °C in the presence of 5% CO_2_. Human MSCs were obtained from Professor Shih-Chieh Hong (Taipei Veterans General Hospital, Taipei, Taiwan) and cultured in alpha-minimum essential medium (α-MEM, Catalog No.: 12561056, Thermo Fisher Scientific, Waltham, MA, USA) supplemented with 20% fetal bovine serum and 1% Penicillin-Streptomycin-Amphotericin B antibiotics. The culture medium was replaced every 2 days and subcultured at 80~90% confluency.

### 2.8. Viability of MG63 Cells and MSCs within the GelMA Composite

MG63 cells and MSCs were embedded within 15% GelMA-based hydrogels for cell culture durations of 1, 7, and 14 days. To produce the staining solution, 5 µL calcein AM and 20 µL ethidium homodimer-1 (Catalog No.: R37601, Thermo Fisher Scientific, Waltham, MA, USA) were added to 10 mL sterile Dulbecco’s PBS (Catalog No.: 14190, Thermo Fisher Scientific, Waltham, MA, USA). The cells were washed with PBS twice and 200 μL of the staining solution was then added directly to culture wells. After 30 min of incubation, fluorescent images were obtained under a fluorescence microscope (BX61, Olympus, Tokyo, Japan).

### 2.9. Cell Proliferation Tests Using MTT Assay

On Day 1, 7, and 14, 3-(4,5-dimethylthiazol-2-yl)-2,5-diphenyl tetrazolium bromide (MTT, Catalog No.: 298-93-1, Sigma-Aldrich, Saint Louis, MO, USA) assay was used to evaluate the proliferation of the MG63 cells and MSCs embedded in the hydrogels. MTT solution (5 mg/mL MTT in sterile PBS) was added to each well and incubated for 4 h at 37 °C. After incubation, the medium was removed from each well and 200 μL dimethyl sulfoxide (Catalog No.: M2128, Sigma-Aldrich, Saint Louis, MO, USA) was added, and then incubated for 30 min with gentle shaking. Test samples (100 μL) from each well were added to a new 96-well plate for measurement of the absorbance at 570 nm using a spectrophotometer (DU640, Beckman Coulter, Brea, CA, USA).

### 2.10. RNA Isolation and Real-Time Reverse Transcription-PCR

The expressions of osteocalcin (OC), osteopontin (OPN), alkaline phosphatase (ALP), and collagen type I, alpha (COL1A1) were analyzed using quantitative polymerase chain reaction (PCR). Total RNA was isolated with Trizol reagent (Catalog No.: 15596026, Thermo Fisher Scientific, Waltham, MA, USA). Briefly, the cell-laden hydrogel was homogenized with 20-G needles and then extracted by chloroform (Catalog No.: C2432, Sigma-Aldrich, Saint Louis, MO, USA) and an RNeasy mini kit (Catalog No.: 74004, Qiagen, Hilden, NRW, Germany). Total RNA (0.5 µg) was reverse-transcribed and then five-fold diluted for PCR amplification (95 °C for 5 s, 58 °C for 15 s, 72 °C for 10 s, 40 cycles). All primers were designed in the following sequence (Table 1) according to the report published by Lin et al. [32].

### 2.11. Statistical Analysis

All experiments were performed in triplicate. Quantitative data were analyzed by one-way analysis of variance (ANOVA). The data were presented as mean ± standard deviation. A value of *p* < 0.05 was considered statistically significant.

## 3. Results and Discussion

### 3.1. Surface Modification of HAp

**Figure 1:**
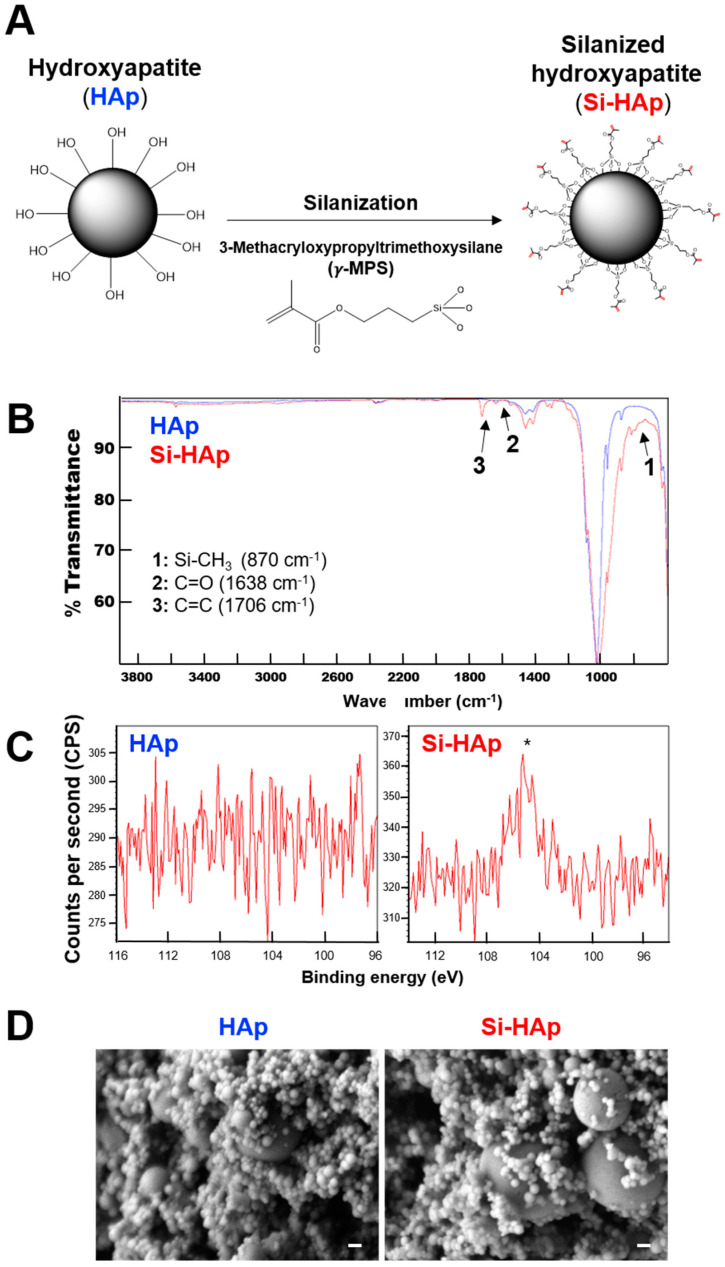
Surface modification of hydroxyapatite. (**A**) Schematic of the silanization of HAp nanopowder using 3-methacryloxypropyltrimethoxysilane. (**B**) ATR-FTIR spectra of HAp and Si-HAp. Characteristic peaks, 1: Si-CH_3_ (870 cm^−1^), 2: C=O (1638 cm^−1^), 3: C=C (1706 cm^−1^). (**C**) X-ray photoelectron spectra of HAp (left) and Si-HAp (right). * Si 2p (Si-O) peak, ~104 eV. (**D**) Scanning electron microscopy images of HAp (left) and Si-HAp (right) particles. magnitude, 50,000×. Scale bar, 100 nm.

First, we attempted to improve the adhesion of HAp fillers to GelMA hydrogels that might increase the stiffness of the hydrogels. To this end, we used a silane coupling agent (*γ*-MPS) to add functional methacrylate groups on the surface of HAp through silanization (Figure 1A). ATR-FTIR analysis was performed to verify whether the hydroxyl groups on the HAp were substituted by methacrylate silane groups. FTIR spectra showed that characteristic peaks of C=C (1638 cm^−1^), C=O (1706 cm^−1^), and Si-CH_3_ (870 cm^−1^) were detected only on the Si-HAp (Figure 1B). The surface elemental compositions of HAp were evaluated by using XPS analysis, and the presence of the peak at approximately 104 eV demonstrated the existence of silicon atoms (Si-oxide) on the Si-HAp (Figure 1C). In addition, the SEM images indicated that the silanization reaction did not alter the surface topography and disturb the spherical structure of HAp and Si-HAp particles (Figure 1D). These data demonstrated that the silanization of HAp was accomplished and that it conjugated the methacrylate groups onto silanized HAp (Si-HAp).

### 3.2. Dispersion of Si-HAp in the GelMA Hydrogel

**Figure 2:**
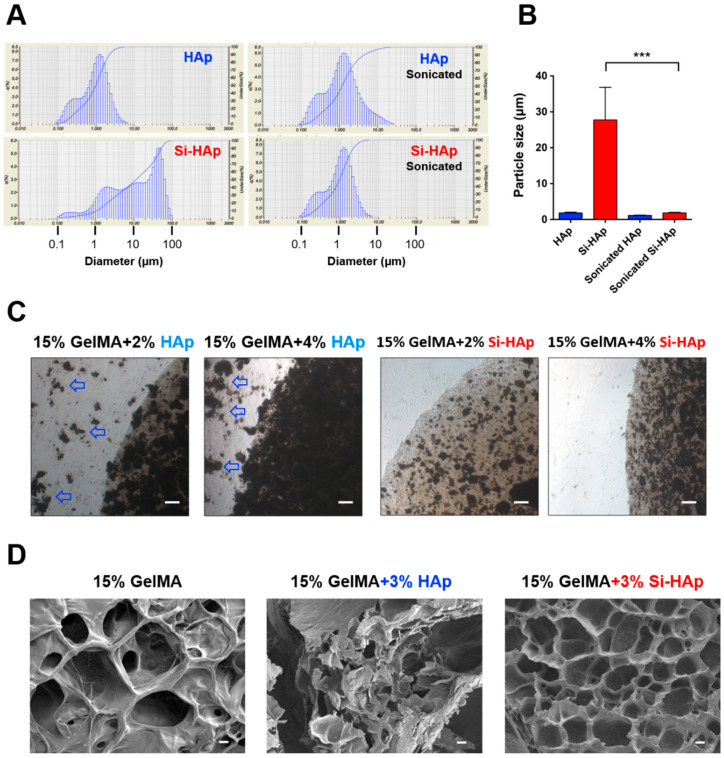
Size of nanoparticle agglomerates and dispersion of hydroxyapatite (HAp) and silanized HAp (Si-HAp) in the gelatin methacrylate (GelMA) solution and composite hydrogels. (**A**) Particle size analysis of HAp particles in the GelMA solution. Left panel: before sonication, Right panel: after sonication. (**B**) Quantification of particle size of HAp and Si-HAp with and without ultrasound sonication. mean ± SD µm. *** *p* < 0.01. (**C**) MG63 cell-embedded composite gels after 24 h incubation. Arrows indicate the debris of unpolymerized GelMA composite gels. Scale bar, 100 µm. (**D**) Scanning electron microscopy images of 15% GelMA, 15% GelMA with 3% HAp and 15% GelMA with 3% Si-HAp composite hydrogels. magnitude, 1000×. Scale bar, 1 µm.

Controlling the dispersion and particle size of nanoparticles in the matrix is crucial to improve the mechanical properties of bulk polymers [35]. Surprisingly, we discovered through particle sizing analysis that Si-HAp powders easily agglomerated and generated larger aggregates in the GelMA pre-gel solution than HAp did. (Figure 2A). The size distribution indicated that the average size of Si-HAp (27.74 ± 7.43 µm) had a >15 fold increase in comparison with the HAp group (1.82 ± 0.18 µm, Figure 2B). Silanization reaction might cause the condensation of HAp particles and facilitate the agglomeration by altering the particle properties. To increase the dispersion and deagglomeration of Si-HAp, we performed the physical method, ultrasound sonication, to generate the stable suspension of HAp and Si-HAp without using a chemical dispersant that may cause cytotoxicity. After sonication, the Si-HAp particles in GelMA solution were more uniform in size and had even dispersion similar to that of HAp in GelMA solution (Figure 2B). Because HAp and Si-HAp can interfere with the light transmission, the amount of fillers added to the hydrogel system became a crucial parameter. Hence, we added HAp or Si-HAp to the 15% GelMA solution from 0–4% (*w*/*v*) to observe the degree of photopolymerization after blue light exposure. The microscopic images showed that hydrogels were unable to photopolymerize completely when the concentration of HAp was increased to 4% (*w*/*v*), whereas the hydrogel was completely crosslinked with 4% Si-HAp under the same light exposure condition. Additionally, hydrogel debris was observed with the concentration of HAp in the 2% and 4% groups (Figure 2C). Moreover, the SEM analysis of the dried GelMA composite hydrogels revealed that the architecture of the 3% HAp hydrogel was more irregular than that of the 3% Si-HAp group and that the porous structure was disorganized in the HAp group (Figure 2D). We inferred that HAp fillers attenuated the crosslinking density and led to a decrease in the mechanical strength of composite hydrogels. Physically mixing unmodified HAp reduced the crosslinking of GelMA hydrogel owing to a lack of covalent binding on its surface. In contrast, Si-HAp in the silanized groups enhanced the bonding between GelMA and fillers, resulting in promoted gelation. Based on these results, we adopted 3% fillers as the optimal ingredient in the subsequent experiments.

### 3.3. Mechanical Properties of Composite Hydrogels

**Figure 3:**
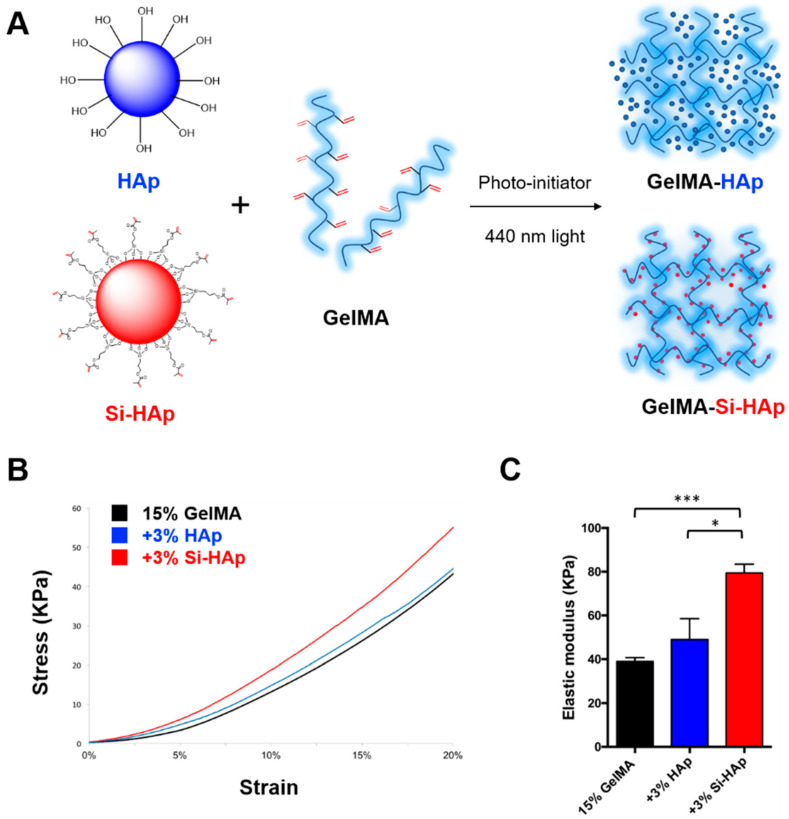
Mechanical properties of cell-free GelMA-HAp and GelMA-Si-HAp composite hydrogels. (**A**) Schematic representation of the preparation of composite hydrogel constructs. (**B**) Stress and strain curve of GelMA-HAp (blue) and GelMA-Si-HAp (red) hydrogels (3% fillers). (**C**) Elastic modulus of GelMA-HAp and GelMA-Si-HAp hydrogels. * *p* < 0.05, *** *p* < 0.001.

GelMA-HAp and GelMA-Si-HAp composite hydrogels were prepared by incorporating 3% HAp or Si-HAp with VA-086 photoinitiator under 440 nm blue light for 1 min (Figure 3A). The C=C double bonds on GelMA and Si-HAp can form covalent bonds through free radical polymerization. To verify the reinforcing effect of silanized fillers, we used the uniaxial compression test to measure the mechanical properties of each composite hydrogel. The stress and strain curve indicated the behavior of the different composite hydrogels, and the elastic modulus was calculated from the slope at 2% strain (Figure 3B). The elastic modulus significantly increased ~2-fold in the 15% GelMA with 3% Si-HAp group in comparison with the pure GelMA hydrogel (from 38.96 ± 1.7 to 79.44 ± 4.0 kPa, *p* < 0.01, Figure 3C). On the basis of these findings, the Si-HAp fillers restored the degree of polymerization and exhibited superior mechanical properties to those of the HAp groups. In summary, the covalent bonding between Si-HAp and GelMA hydrogel enhanced the stiffness of photo-crosslinking hydrogels.

### 3.4. Cell Encapsulation in the GelMA–Si-HAp Hydrogel

**Figure 4:**
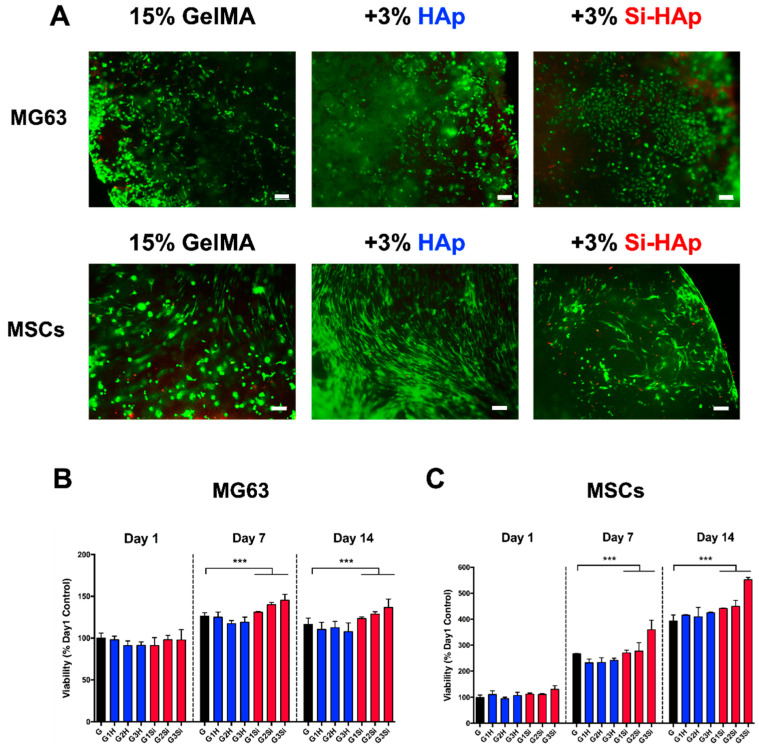
MG63 cells and human mesenchymal stem cells (MSCs) encapsulation by GelMA composite hydrogels. (**A**) Live/dead cell viability assay of human MG63 cells and MSCs on day 14. green, calcein AM, red, ethidium homodimer-1. Scale bar, 100 µm. (**B**) MTT assay of human MG63 cells on day 1, day 7, day 14. (**C**) MTT assay of human MSCs on day 1, day 7, day 14. (G: 15% GelMA, G1H-G3H: 15% GelMA with 1–3% HAp, G1Si–G3Si: 15% GelMA with 1–3% Si-HAp). *** *p* < 0.001.

An appropriate hydrogel for tissue engineering can encapsulate various cell types and mimic a 3D microenvironment to support cell growth [36]. We encapsulated the osteoblast-like MG63 cells and human MSCs in HAp and Si-HAp composite hydrogels to assess the cytotoxicity of these photocrosslinkable hydrogels. The cell viability assay revealed that embedded cells were almost alive (green) in all groups after 14 days of cell culture (Figure 4A). Cell proliferation was evaluated using MTT assay and the results indicated that MG63 and MSCs embedded in 3% Si-HAp GelMA hydrogels exhibited the highest proliferation on day 14 (Figure 4B,C). These data demonstrated that the composite hydrogel had no cytotoxic effects and maintained cell viability in all groups regardless of the amounts of fillers that were incorporated.

### 3.5. Osteogenic Differentiation of Encapsulated Cells in the GelMA–Si-HAp Hydrogel

**Figure 5:**
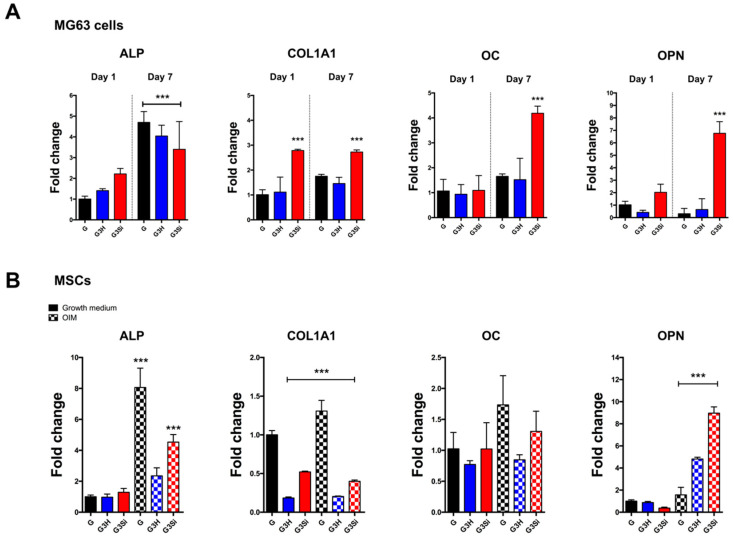
Gene expression of osteogenic markers in cell-laden GelMA composite hydrogels. (**A**) The expressions of alkaline phosphatase (ALP), collagen type 1, alpha 1 (COL1A1), osteocalcin (OC), and osteopontin (OPN) in MG63 cell-laden composite hydrogels were quantified by qPCR on day 1 and day 7. Fold change was normalized to the day 1 GelMA group. (**B**) Four osteogenic markers in MSCs laden composite hydrogels were quantified by qPCR on day 7. MSCs were cultured with growth medium or osteogenic induction medium. Gene expression was normalized to the GelMA group with growth medium. G: 15% GelMA, G3H: 15% GelMA with 3% HAp, G3Si: 15% GelMA with 3% Si-HAp. *** *p* < 0.01.

To achieve the aim of bone regeneration, we developed the composite hydrogel not only for cell delivery to the tissue damaged sites but also provided an in vivo like microenvironment to support cell differentiation. To further investigate whether these composite hydrogels can promote the osteogenic differentiation of encapsulated cells, the MG63 cells and MSCs were embedded in the composite gels. After 7 days of cell culture, real-time quantitative PCR analyses (qPCR) were conducted to detect the expression of four osteogenic markers (ALP, COL1A1, OC, and OPN). The qPCR results of MG63 encapsulated hydrogels indicated that the expression of ALP was increased in all groups on day 7 and that no significant changes were observed between the HAp and Si-HAp hydrogels. The expression of COL1A1 was increased in the GelMA–Si-HAp hydrogel on day 1 and 7, and the expression of OC and OPN were increased in GelMA-Si-HAp on Day 7 (Figure 5A). Addition of Si-HAp (3%) in the MG63 encapsulating GelMA hydrogel had a higher expression of COL1A1, OC, and OPN in comparison with the HAp groups. For MSCs encapsulating hydrogels after osteogenic induction, the expression of ALP was increased in both the GelMA and GelMA-Si-HAp groups on day 7 but had no significant difference in GelMA-HAp. The expression of COL1A1 and OC exhibited no significant increase in the composite hydrogels. The GelMA-Si-HAp group had the highest expression of OPN on day 7 (Figure 5B). The results indicated that GelMA-Si-HAp had greater effects on osteogenic differentiation in MG63 cells than in MSCs. These data also indicated that GelMA-HAp attenuated the expression of some osteogenic markers (ALP, COL1A1, and OC) compared with the Si-HAp groups in both MG63 and MSCs encapsulated hydrogels. In conclusion, we found that the GelMA composite hydrogel can enhance bone differentiation in osteoblast-like MG63 and that the addition of silanized HAp in the GelMA has superior enhancing effects to those of the GelMA-HAp composite.

### 3.6. Photolithography of the GelMA-Si-HAp Composite Hydrogels with Visible Light

**Figure 6:**
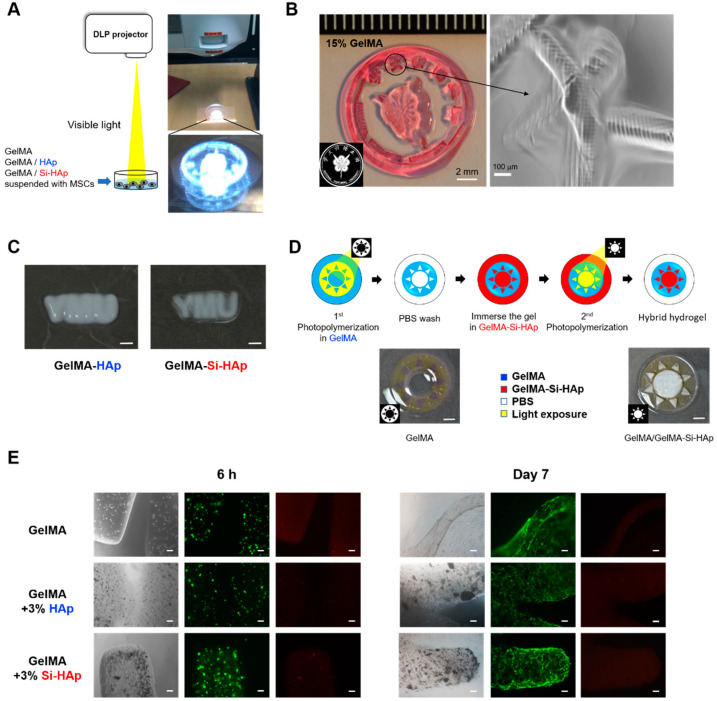
GelMA composite hydrogels were fabricated by visible light projection. (**A**) Schematic of the photolithographic method that was to construct GelMA-based hydrogels with a digital light processing (DLP) projector. (**B**) 15% cell-free GelMA hydrogel fabricated into the logo of National Yang-Ming University by projection (stained with neutral red). Scale bar, 2mm. Right, the hydrogel was observed through optical microscopy. Scale bar, 100 µm (**C**) 15% GelMA with 3% HAp and GelMA with 3% Si-HAp composite hydrogels developed using the DLP-based projector for 40 s). Scale bar, 5 mm. (**D**) Sequential fabrication of GelMA and GelMA-Si-HAp composite hydrogels in a hybrid pattern. Scale bar, 5 mm. (**E**) Cell viability assay of MSCs encapsulated in hydrogels through photolithography after 6 h and 7 days of incubation. green, calcein AM, red, ethidium homodimer^−1^. Scale bar, 100 µm.

To fabricate a photo-patterned hydrogel, we established a simple and rapid system to perform optical lithography for the GelMA hydrogel by using a common DLP-based projector under white light illumination. To assemble the hierarchical structure of the cell encapsulating hydrogel, we first developed a photocrosslinking method of GelMA hydrogels by using a commercial projector under visible light (Figure 6A). The logo of National Yang-Ming University was constructed with GelMA hydrogel, and the microscopic images displayed the pixelated texture of the engineered hydrogel, which was created by light reflection from the digital micromirror device (Figure 6B). Next, we used the HAp and Si-HAp based GelMA solution to generate the letters “YMU” (Figure 6C). The image depicts that the resolution produced by GelMA-HAp was lower than GelMA-Si-HAp due to its higher lateral diffraction and bulging shape. We identified that GelMA-Si-HAp solution exhibited low lateral diffraction and had the ability to polymerize in a shorter time than the HAp composite hydrogel could, serving as an effective filler in the GelMA system. To construct the hybrid hydrogel, we assembled two different GelMA-based hydrogels by curing the first hydrogel into a circle shape and washing the unpolymerized solution. We then cured the second hydrogel onto the remaining area using a DLP-based projector (Figure 6D). To detect the viability of cell-embedded hydrogels that were produced by the projection system, the live and dead assay of MSCs was performed after both 6 h and 7 days of incubation. Most of encapsulated MSCs were alive and had spread in the composite hydrogel after 7 days (Figure 6E). On the basis of the aforementioned data, we suggest that visible light projection can be used to fabricate the complex 3D architecture of GelMA hydrogels without causing cytotoxicity.

In the present study, we selected the silanized HAp (Si-HAp) as an additive in the hydrogel system. After surface modification, the surface composition of the nanoscale HAp powders was examined by using Fourier transform infrared (FT-IR) spectroscopy, x-ray photoelectron spectroscopy (XPS), and scanning electron microscopy (SEM). The data demonstrated that the silane groups were conjugated on the surface of Si-HAp nanoparticles. We analyzed the particle size in the GelMA solution to examine the dispersion of nanoparticles The good dispersion of HAp and Si-HAp is required to construct the composite hydrogel. The data indicated that the composite GelMA hydrogel with 4% Si-HAp was completely polymerized after light exposure. In addition, the mechanical properties of the composite hydrogels were measured using compression tests to evaluate the reinforcing effect of Si-HAp. The elastic modulus significantly increased over two-fold in 15% GelMA composite hydrogel with 3% Si-HAp. The cell viability test and MTT assay of MG63 and MSCs indicated that the embedded cells had high cell viability after 14 days. The osteogenic differentiation of the encapsulated osteoblasts and mesenchymal stem cells was also evaluated by qPCR to demonstrate that GelMA composite hydrogel can enhance bone regeneration. Furthermore, we developed a visible light curing system by using a commercial DLP-based projector to micro-fabricate these composite hydrogels into complex architecture. The results revealed that the resolution produced by GelMA-HAp was lower than GelMA-Si-HAp due to its higher lateral diffraction. We identified that GelMA-Si-HAp solution exhibited low lateral diffraction and had the ability to polymerize in a shorter time than the HAp composite hydrogel could.

The silanization of fillers has demonstrated the potential to improve the crosslinking of synthetic polymers and further enhance the physical properties [37,38,39,40]. Methacrylate groups on fillers are able to form covalent bonds between polymers and fillers after polymerization [41]. In this study, the reinforcing effect of incorporated Si-HAp in the GelMA hydrogel was first verified. Consistent with other reports, the silanization reaction of filling materials is crucial to reinforce the hydrogel through methylacrylate groups on the fillers. In 2014, Cha C. et al. reported that the addition of methacrylate graphite oxide in the GelMA hydrogel resulted in enhanced stiffness [42]. In the present study, we selected HAp as a filler in GelMA hydrogels because it is a natural inorganic mineral in human bone, and because HAp nanoparticles have been used for decades in clinical practice. In addition, nanoscale synthetic HAp exhibits more therapeutic efficacy than microscale HAp does due to its unique physical properties. In particular, a large surface area of fillers increases the efficiency of silanization and forms more covalent bonds on the surface of HAp during polymerization. Our data suggested that silanization of HAp is a promising and safe method to improve the material properties of cell-laden GelMA hydrogels. Although HAp and gelatin have been approved by Food and Drug Administration, the use of silanized HAp and methacrylate gelatin might be a fast and applicable strategy in clinics.

For cell delivery, this composite hydrogel system has been demonstrated to be a biocompatible material for osteoblast and MSC encapsulation (Figure 4). We, therefore, hypothesized that adding the osteoconductive HAp to the GelMA hydrogel would further promote MSCs differentiation into bone cells after osteogenic induction. However, only some osteogenic markers were increased on day 7 in MSCs (Figure 5). We speculated that the physical properties of experimental hydrogels were unable to facilitate encapsulated cell differentiation. MSC differentiation demonstrably depends on the matrix elasticity of the surrounding microenvironment [43]. To improve the osteogenic differentiation of cells in our hydrogel system, the matrix stiffness must be tuned within a physiologically relevant range by adjusting the amount of fillers and GelMA. Moreover, the degradation of hydrogels influenced the gene expression of osteogenic markers, especially on day 7, when the integrity of the hydrogel was broken resulting in a low RNA yield that might have influenced the qPCR results. Furthermore, the hydrolysis rate of HAp is relatively slower than those of other calcium phosphate salts, replacing HAp with beta-tricalcium phosphate is another possible strategy for improving cell differentiation. In summary, the optimized conditions should be identified in the future to enhance and prolong the effects of cell differentiation.

Because the fillers are opaque materials that can attenuate light transmission, their particular properties become crucial parameters for controlling the degree of photopolymerization. In this study, the Si-HAp used in the GelMA system did not affect photo-crosslinking, and the composite hydrogel could be completely crosslinked with a concertation of Si-HAp over 4% (*w*/*v*). In addition, the choice of light source improved the light transmission by using longer wavelength light than UV, and UV rays have more difficulty penetrating the composite hydrogel solution than visible light has. Thus, we selected visible light as a light source to form thick hydrogels. With the aid of DLP-based projection, we used a digital micro-mirror device to control the exposed area of visible light and thereby construct the 3D cell-laden hydrogels in the complex shape. Currently, DLP-based projectors are commercially available, and the simple projection system we reported on in this study can be easily assembled by researchers. On the basis of these findings, we further developed a bioprinting method with a motorized x-y-z stage and a DLP projector to fabricate the 3D cell-laden hydrogels layer-by-layer. In the future, we will optimize the system to produce a cost-effective 3D bioprinter for biomaterial applications.

Investigating the relationship between the GelMA-Si-HAp hydrogel and bone is critical to evaluate the new bone formation. It has been previously reported that histological sections of GelMA scaffold taken after 12 weeks from a critical-size rat calvarial bone defect site showed increased regeneration of defective bone [44]. In vitro imaging of the GelMA scaffold has been performed to evaluate bone regeneration by using micro-computed tomography [45]. A histologic study reported that MSC-loaded nano-HAp exhibited higher bone formation at 6 and 12 weeks after operation [46]. In this study, we selected the GelMA hydrogel with MSCs and Si-HAp nanoparticles as a reinforced scaffold to promote bone regeneration. Due to the lack of histologic data, in vivo research is needed to establish in the future. Moreover, the mineralization of polymeric hydrogels is required to enhance the regenerative potential of hydrogels [47]. The calcium phosphate precipitated on the hydrogel scaffold surface after immersion in simulated body fluid was exanimated by X-ray Diffraction (XRD) analysis [48]. Therefore, the formation of an apatite layer on the surface of the composite hydrogel is crucial to evaluate the potential of bone regeneration. In future research, more research is needed to apply and test the mineralization potential of the composite hydrogel.

## 4. Conclusions

We considered a reinforced and photocrosslinkable composite hydrogel incorporated with Si-HAp. The mechanical properties of the composite hydrogels were enhanced by the covalent bonding between the interface of HAp nanoparticles and GelMA. In addition, this hybrid hydrogel system demonstrated high biocompatibility for osteoblast-like cells and MSC encapsulation and promoted osteogenic differentiation in osteoblasts. A reinforced hydrogel can be produced by visible light polymerization and form complex architecture constructs by using the simple projection system. These findings support the use of this composite hydrogel as a design biomaterial in bone tissue engineering.

## Figures and Tables

**Table 1:** Sequences of primers used for qPCR.

Gene	Primer Sequence
GAPDH	5′-forward-GGAGCGAGATCCCTCCAAAAT
	5′-reverse-GGCTGTTGTCATACTTCTCATGG
COL1A1	5′-forward-GAGGGCCAAGACGAAGACATC
	5′-reverse-GGCTGTTGTCATACTTCTCATGG
Osteocalcin	5′-forward-GAGGGCCAAGACGAAGACATC
	5′-reverse-CCCTCCTGCTTGGACACAAAG
OPN	5′-forward-CTCCATTGACTCGAACGACTC
	5′-reverse-CAGGTCTGCGAAACTTCTTAGAT

## Data Availability

Data is contained within the article.
